# Sound, Force and Light Induced Emissions from Er^3+^‐Mn^2+^ Doped ZnS/CaZnOS Heterostructure for Remote Temperature Monitoring via Photo‐ and Mechanoluminescence

**DOI:** 10.1002/adma.202510117

**Published:** 2025-07-23

**Authors:** Marcin Runowski, Jan Moszczyński, Przemysław Woźny, Kevin Soler‐Carracedo, Justyna Barzowska, Sebastian Mahlik, Dengfeng Peng, Teng Zheng

**Affiliations:** ^1^ Faculty of Chemistry Adam Mickiewicz University Uniwersytetu Poznańskiego 8 Poznań 61‐614 Poland; ^2^ School of Information and Electrical Engineering Hangzhou City University Hangzhou 310015 China; ^3^ Institute of Experimental Physics Faculty of Mathematics Physics and Informatics University of Gdansk Wita Stwosza 57 Gdansk 80‐308 Poland; ^4^ Shenzhen Key Laboratory of Intelligent Optical Measurement and Detection Shenzhen University Shenzhen 518060 China; ^5^ Key Laboratory of Optoelectronic Devices and Systems of the Ministry of Education and Guangdong Province College of Physics and Optoelectronic Engineering Shenzhen University Shenzhen 518060 China; ^6^ State Key Laboratory of Radio Frequency Heterogeneous Integration Shenzhen University Shenzhen 518060 China

**Keywords:** lanthanide ions, luminescence thermometry, sound‐to‐light conversion, temperature sensors, visual sensors of force and sound

## Abstract

Mechanoluminescence (ML) is a powerful phenomenon that enables light generation induced with mechanical or acoustic waves, and remote temperature sensing via luminescence thermometry techniques. In this work, the multi‐functional, ML‐active materials based on Er^3+^ and Mn^2+^ co‐doped ZnS/CaZnOS heterostructure are developed for remote temperature monitoring and visual sensing of force and sound. The material exhibits characteristic photoluminescence (PL) under UV and NIR (up‐conversion) excitation, with energy transfer from Er^3+^ to Mn^2+^ influencing the emission color. The effects of force‐to‐light conversion are studied in detail by measuring the ML intensity versus the applied power for Er^3+^ and Mn^2+^ emission in the single‐doped and co‐doped materials. Temperature‐dependent PL is utilized to calibrate luminescence thermometry response, with Er^3+^ thermally‐coupled levels and non‐thermally‐coupled levels of Er^3+^/Mn^2+^, providing temperature sensing capabilities. The unique combination of sound‐induced ML with luminescence thermometry allowed optical temperature detection, alike during the drilling process, and in the externally heated system, using pulsed sonications. Whereas, applying continuous excitation, the sound‐to‐heat conversion is studied and visualized using the developed ML‐based optical thermometers. This approach demonstrates the excellent application potential of sound‐to‐light conversion for remote monitoring and, more importantly, for excitation‐light‐free temperature probing of different systems and working devices.

## Graphical abstract

Er^3^⁺/Mn^2^⁺ co‐doped ZnS/CaZnOS heterostructures are developed for remote temperature and force/sound sensing. The material exhibits UV/NIR photoluminescence, with energy transfer influencing color. Mechanoluminescence intensity is measured against applied power. Temperature‐dependent photoluminescence enabled luminescence thermometry. Sound‐induced mechanoluminescence, combined with thermometry, facilitated temperature detection during drilling and in heated systems. This demonstrates potential for excitation‐light‐free temperature probing.

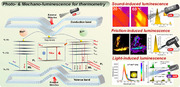

## 1 Introduction

Mechanoluminescence (ML) is a physical phenomenon characterized by the emission of light in response to mechanical force on a solid material.^[^ ^1^, ^2^, ^3^, ^4^, ^5^, ^6^, ^7^ ^]^ Typical ML materials can generate light triggered by mechanical forces encompass various forms, including stretching, compression, bending, friction, and impact.^[^ ^2^, ^8^, ^9^, ^10^, ^11^ ^]^ Sound waves, as a form of mechanical energy transfer, can transmit energy through molecular/atom vibrations, penetrating solid, liquid media, or biological tissues as longitudinal or transverse waves, and efficiently deliver energy to target locations.^[^ ^12^, ^13^ ^]^ Compared to traditional ML materials, the sound‐induced ML in piezoelectric materials demonstrate many benefits, such as: I) real‐time excitation of light without direct surface contact, avoiding large displacements and minimizes the risk of matrix damage or interference from friction or mechanical interaction, which grants superior application potential for inspecting precision or fragile materials; II) high‐efficiency energy transfer, enabling more uniform material excitation and significantly higher luminescence efficiency than traditional contact‐based methods; III) parameters such as frequency and amplitude of ultrasonic waves can be flexibly adjusted to precisely control the intensity and properties of ML, which offer enhanced adaptability and operability for diverse application scenarios;^[^ ^12^, ^14^, ^15^ ^]^ IV) ultrasonic waves show superior penetration depth (e.g. deep tissue penetration exceeding 10 cm) and effectively excite ML in diverse substrates, including metals, ceramics, composites, and deep biological tissues, surpassing the limited scope of traditional contact‐based ML materials.^[^ ^16^, ^17^, ^18^, ^19^, ^20^ ^]^ Additionally, they have advantages like excellent biosafety, high spatiotemporal resolution, rapid response, real‐time operation, low cost, and wide applicability. The sound‐induced ML emerges as an unique and modern tool in noninvasive neuronal modulation, targeted activation of cancer immunotherapy, bio‐imaging, sensing, photocatalysis, photobioreactors, optogenetics, and photonic human–machine interfaces.^[^ ^16^, ^17^, ^18^, ^19^, ^20^, ^21^ ^]^ Despite its tremendous potential, the related studies remain at their early stages, requiring further research.

Luminescence thermometry, as a modern non‐contact method for optical temperature detection, typically utilizes phosphor materials doped with lanthanide ions, utilizing their characteristic narrow emission bands originating from the intra‐configurational 4f–4f transitions, and the appropriate thermally‐coupled levels (TCLs).^[^ ^7^, ^22^, ^23^, ^24^ ^]^ Combination of the TCLs of selected lanthanide ions (e.g. Er^3^⁺, Tm^3^⁺, or Nd^3^⁺) with luminescence intensity ratio (LIR) method, allows facile, rapid, accurate, and remote temperature detection in the systems studied, ensuring good spatial resolution and precise thermal readouts.^[^ ^7^, ^25^, ^26^, ^27^, ^28^, ^29^, ^30^, ^31^ ^]^

The use of sound‐induced ML is a truly novel and powerful strategy for next‐generation optical thermometry applications, showcasing superior advantages like large penetration depth, lack of laser‐induced heating and tissue damage, less electromagnetic interferences, excitation through the non‐transparent surfaces (e.g. skin or metal), etc.^[^ ^17^, ^18^, ^20^ ^]^ On the other hand, stress/force sensing has received significant research interest across various fields, including wearable devices, robotics, and flexible electronics, evolving into a prominent subdiscipline within advanced materials and manufacturing.^[^ ^15^, ^32^, ^33^ ^]^ Several works have shown that ML intensity exhibits a strong correlation with applied stress, rendering it particularly suitable for stress‐sensing applications.^[^ ^5^, ^34^, ^35^ ^]^ Notably, ML‐based sensing offers distinct advantages by addressing the limitations inherent in conventional sensing technologies, thereby facilitating novel applications in emerging domains.^[^ ^5^, ^9^, ^36^, ^37^, ^38^, ^39^, ^40^ ^]^

Here, we developed multifunctional ML‐active materials based on Er^3^⁺ and Mn^2^⁺ co‐doped ZnS/CaZnOS heterostructures for remote temperature monitoring and visual force/sound detection. Under UV and NIR (up‐conversion) excitation, the materials exhibit tunable photoluminescence (PL) mediated by energy transfer from Er^3^⁺ to Mn^2^⁺. The force‐ and sound‐to‐light conversions were systematically investigated by analyzing ML intensity versus the magnitude of the applied stimuli for Er^3^⁺ and Mn^2^⁺ emissions, in single‐doped and co‐doped systems. The optical thermometric response of the materials studied was used for the PL‐ and ML‐based temperature monitoring. By using sound‐induced ML for optical sensing purposes, we demonstrate remote temperature probing in the externally heated systems using pulsed sonication. In other scenarios, by using friction‐induced ML we show temperature elevation during the drilling processes. Continuous focused‐ultrasound excitation further enables visualization of sound‐to‐heat conversion via ML‐based thermometry. This strategy highlights the potential of ML for remote and excitation‐light‐free temperature probing in operational devices and dynamic systems.

## 2 Results and Discussion

### 2.1 Basic Structural and Spectroscopic Properties

In order to enhance the ML intensity of the Er^3+^ and Er^3+^‐Mn^2+^ doped CaZnOS samples, we fabricated them in the form of the well‐established heterostructure material ZnS/CaZnOS, composed of ZnS and CaZnOS phases,^[^ ^41^, ^42^ ^]^ at a molar ratio 3:2 (see the synthesis details in the Supporting Information). This is because, according to previous reports, the ZnS component improves the susceptibility of the CaZnOS:Mn^2+^/Ln^3+^ (Ln = lanthanide ions) particles to mechanical stimuli, resulting in a significantly boosted ML signal from the final heterostructure‐type product ZnS/CaZnOS:Mn^2+^/Ln^3+^.^[^ ^41^, ^42^ ^]^

**Figure 1:**
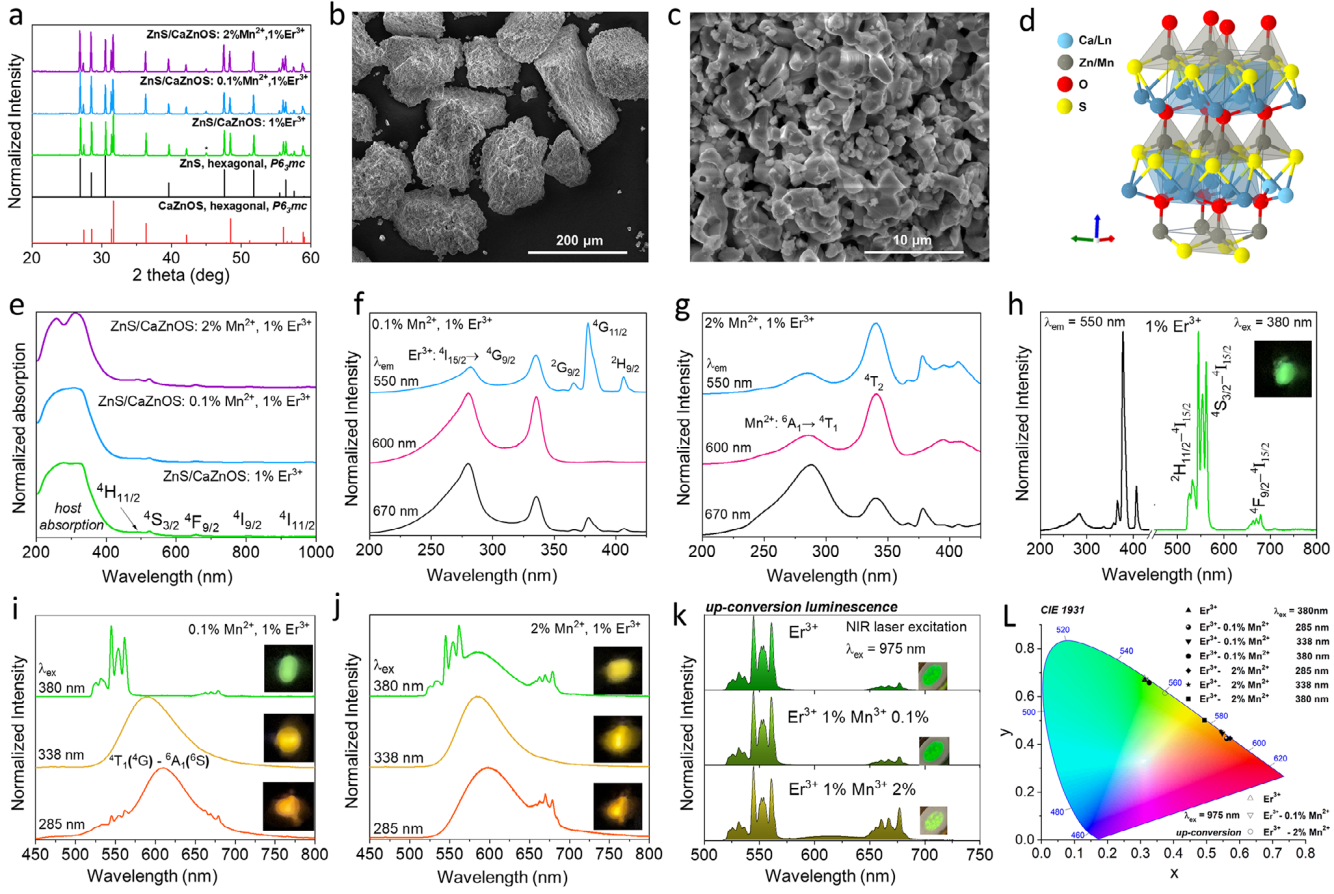
a) Powder XRD patterns of the synthesized heterostructure materials ZnS/CaZnOS doped with Er^3+^ and co‐doped with Er^3+^‐Mn^2+^ ions. b,c) Representative SEM images at different magnifications for the sample ZnS/CaZnOS: 1% Er^3+^‐ 2% Mn^2+^. d) 3D representation of the investigated crystal structure. e) Absorption spectra for the synthesized materials, recorded in a diffuse‐reflectance mode. f,g) Excitation spectra for the ZnS/CaZnOS co‐doped with 1% Er^3+^ and 0.1% Mn^2+^ or 2% Mn^2+^, recorded at varied emission wavelengths (λ_em_ = 550, 600, and 670 nm). h) excitation (λ_em_ = 550 nm; black curve) and emission (λ_ex_ = 380 nm; green curve) spectra for the ZnS/CaZnOS doped with 1% Er^3+^. i,j) Emission spectra for the materials co‐doped with 1% Er^3+^ and 0.1% Mn^2+^ or 2% Mn^2+^, measured at different excitation wavelengths (λ_ex_ = 285, 338 and 380 nm). k) Up‐conversion emission spectra (λ_ex_ = 975 nm NIR laser; flux ≈5 W/cm^2^) recorded for all samples. l) Chromaticity diagram (CIE 1931) with displayed color coordinates for the materials studied, based on the corresponding emission spectra acquired at different λ_ex_ values; the insets presented (h‐k) display the photographs of the materials' PL colors, taken under irradiation with the indicated λ_ex_.

The powder X‐ray diffraction (XRD) patterns of the obtained ZnS/CaZnOS: Er^3+^ (1 mol%), ZnS/CaZnOS: Er^3+^‐Mn^2+^ (1: 0.1 mol%) and ZnS/CaZnOS: Er^3+^‐Mn^2+^ (1: 2 mol%) presented in **Figure** **1**a, confirm the desired ML‐active hexagonal structure and space group *P*63*mc* for both phases, as they fit well with the corresponding reference patterns, i.e., JCPDS 00‐001‐0677 for ZnS and JCPDS 04‐011‐1217 for CaZnOS. The additional reflex marked with an asterisk corresponds to the inert CaS phase, i.e., impurity formed during the synthesis process, which has a negligible effect on the ML performance of the final materials. Figure 1b,c show the representative SEM images of the ZnS/CaZnOS: 1% Er^3+^‐ 2% Mn^2+^ material, revealing that it consists of irregular, individual micron‐sized particles (size ≈1–5 µm), forming larger aggregates (≈100–200 µm), which is typical for these materials synthesized via high‐temperature solid‐state method.^[^ ^41^, ^43^ ^]^ A 3D representation of the CaZnOS structure, with indicated substitution of the dopant ions, is depicted in Figure 1d. It is clear that due to the similar ionic radii of Ca^2+^ and Er^3+^, as well as Zn^2+^ and Mn^2+^, erbium (III) ions substitute calcium ions, whereas manganese (II) ions substitute zinc ions in their crystallographic positions, as was already confirmed by the previous literature reports.^[^ ^41^, ^43^ ^]^

The absorption spectra of the synthesized material shown in Figure 1e reveal a strong, broad absorption band of the host below ≈400 nm, which superimposed on the manganese (II) absorption in the case of the highly‐doped sample ‐ 2% of Mn^2+^ (top spectrum). Moreover, a series of characteristic, narrow absorption bands of Er^3+^, originating from its 4f–4f transitions (e.g., ^4^I_15/2_ → ^4^H_11/2_, ^4^S_3/2_, ^4^F_9/2_, ^4^I_9/2_, ^4^I_11/2_,) can be observed in the entire spectral range for all samples. Additionally, the recorded EDX spectra (see Figure S1, Supporting Information) confirm the presence of the dopant Er^3+^ and Mn^2+^ ions, as well as the host elements Zn, Ca, O, and S.

### 2.2 Photoluminescence (PL) Properties

The excitation spectra for the Er^3+^‐Mn^2+^ co‐doped samples, recorded at different monitoring wavelengths–λ_em_ (Figure 1f,g), indicate a significant impact of the λ_em_ on the spectra shape. This is because, recording the spectra at λ_em_ = 550 nm (top curves), the Er^3+^ green PL is mainly monitored, whereas at λ_em_ = 600 nm (central parts), we monitor mainly the Mn^2+^ orange PL. Note that in the latter case, the elevated intensity of the band peaked ≈280 nm (for 0.1% of Mn^2+^), and the characteristic, structured band ≈400 nm (for 2% of Mn^2+^) reveals the energy transfer from Er^3+^ to Mn^2+^ ions. Whereas, for the λ_em_ = 670 nm (bottom), the contribution of both emitting ions is observed i.e., Er^3+^ and Mn^2+^ are excited as their emissions overlap at around 670 nm. The characteristic bands of Er^3+^ and Mn^2+^ were assigned to the corresponding transitions of these ions in Figure 1f,g, respectively, i.e. for Er^3+^: ^4^I_15/2_ → ^4^G_9/2_ (283 nm), ^4^I_15/2_ → ^2^G_9/2_ (366 nm), ^4^I_15/2_ → ^4^G_11/2_ (380 nm) and ^4^I_15/2_ → ^2^H_9/2_ (407 nm) labeled in Figure 1f; and for Mn^2+^: ^6^A_1_ → ^4^T_1_ (286 nm) and ^6^A_1_ → ^4^T_2_ (340 nm). The correct assignment of the bands is confirmed by the excitation spectrum for the single‐doped sample ZnS/CaZnOS: 1% Er^3+^ (without manganese ions), where only the characteristic Er^3+^ bands are present, without contribution of the broad band ≈340 nm associated with Mn^2+^ (Figure 1h; left–black curve).

The emission spectra for all samples, recorded at different excitation wavelengths–λ_ex_ (Figure 1h–k), with a xenon lamp as a light source, indicate substantial effect of the λ_ex_ used on the shape of the emission spectra, as well. Considering the UV light excitation (Figure 1h–j), the use of λ_ex_ = 380 nm results in dominant, green emission of Er^3+^, and for the highly‐doped sample (2% of Mn^2+^) there is also some contribution of Mn^2+^ band, resulting in the yellow like emission, confirming again the energy transfer from Er^3+^ to Mn^2+^. On the other hand, using 338 nm excitation, only the broad band centered ≈590 nm, originating from Mn^2+^ emission, i.e. ^4^T_1_(^4^G) → ^6^A_1_(^6^S) transition, is observed for both concentrations of Mn^2+^, revealing in this case the negligible contribution of the back‐energy transfer from Mn^2+^ to Er^3+^. Whereas, for the λ_ex_ = 285 nm, the dominant, broad band of Mn^2+^ (centered at ≈600 nm), superimposes with less intense, narrow emission bands of Er^3+^, leading to the orange PL of the materials studied. Interestingly, the presence of Mn^2+^ enhances red emission of Er^3+^ (≈670 nm), at the expense of its green emission bands, as clearly seen for the highly‐doped sample (2% of Mn^2+^). This observation implies the presence of some back energy transfer in the highly‐doped material, which occurs predominantly at 285 nm excitation (in contrast to the use of λ_ex_ = 338 nm), indicating different site occupation for Mn^2+^ ions in the heterostructure material studied. It is worth noting, that the use of higher energy excitation wavelength (285 nm), leads to the red‐shift of the emission band (by ≈15‐20 nm). The impact of excitation wavelength on the spectral position of the emission band centroid is associated with the presence of different sites for Mn^2+^ ions in the investigated heterostructure materials, having slightly altered energies of the emitting states, as shown in the previous literature reports.^[^ ^41^, ^42^ ^]^ Whereas, the use of high doping concentration causes an opposite effect, i.e., a slight blue‐shift (by ≈5–10 nm) for the given λ_ex_.

Additionally, we used the NIR 975 nm laser excitation to check the potential up‐conversion emission of the synthesized materials. Surprisingly, even at very low laser power density, i.e. ≈5 W cm^−2^, which is a minimal value for the nonlinear process of energy up‐conversion (see the non‐normalized spectra, laser‐power dependence, slopes values and the related discussion in the Figures S2 and S3, Supporting Information), we could clearly see characteristic narrow emission bands of Er^3+^ in the spectra (Figure 1k) and green emission of the samples. For the sample with 2% of Mn^2+^, one can notice a minor contribution of Mn^2+^ broad‐band emissions, which supports the postulated Er^3+^→Mn^2+^ energy transfer, affecting the final PL color. The insets in Figure 1h–k show the bright PL of the materials upon a given light UV excitation h‐j) and under NIR laser irradiation k) for the up‐conversion emission. An apparent effect of material compositions and excitation wavelengths on the resulting emission colors, from green to yellow and orange, can be observed. Based on the recorded emission spectra under UV and NIR excitations, we determined the color coordinates for all λ_ex_ used and the materials studied, as displayed in the chromaticity diagram (CIE 1931) in Figure 1L. The calculated color coordinates agree well with the PL colors observed by the naked eye and photographs taken with a digital camera, revealing the shift from green via yellow to orange emission color, along with a decreasing value of λ_ex_ and adding Mn^2+^ ions.

### 2.3 PL Versus Force‐ and Sound‐Induced ML

**Figure 2:**
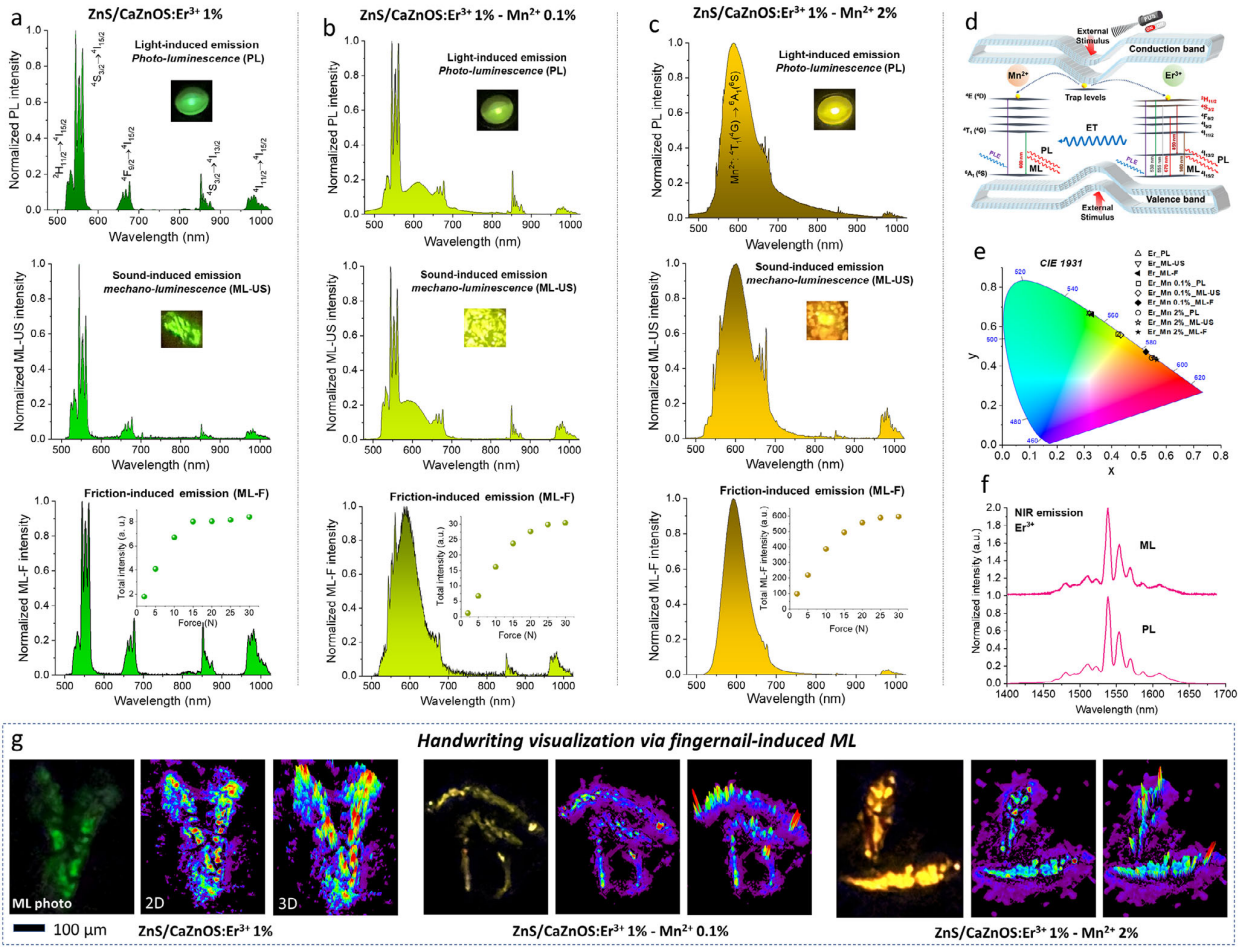
a–c) PL (top), sound‐induced ML (center), and friction‐induced ML spectra (bottom) for the Er^3+^ single‐doped sample, and co‐doped with 0.1% of Mn^2+^ or 2% of Mn^2+^. The insets presented (a‐c) display photographs of the materials' luminescence colors, taken under different excitation modes, i.e., PL‐light (top), ML‐sound (center), and ML‐force (bottom). d) Scheme of the proposed mechanism for PL and ML phenomena in the heterostructure materials studied‐ZnS/CaZnOS doped with Er^3+^ and Mn^2+^. e) Chromaticity diagram (CIE 1931) with displayed color coordinates for the materials studied, based on their luminescence generated by different stimuli, i.e., light (PL), sound (ML), and force (ML). f) NIR emission spectra for the Er^3+^‐doped compound, recorded in PL and ML modes. g) Handwriting visualization via fingernail‐induced ML: photographs of the investigated materials' ML colors (left in a series), as well as the corresponding 2D (center) and 3D (right) images, obtained upon transformation of their intensities into numerical matrices.

**Figure** **2** presents comparison of PL and ML spectra for all samples (a–Er^3+^; b–Er^3+^/Mn^2+^ 0.1%; c–Er^3+^/Mn^2+^ 2%), recorded in a broad spectra range (475–1025 nm), using electromagnetic wave, i.e. a 375 nm UV laser excitation (top), mechanical wave, i.e. a focused ultra‐sound source (center) and a mechanical impact (bottom), as triggers for light generation. First, despite the excitation source, the spectra for the same materials resemble each other, especially for the single‐doped material ‐ Er^3+^ (a), displaying a series of narrow bands corresponding to the transitions of Er^3+^: ^2^H_11/2_ → ^4^I_15/2_ (530 nm), ^4^S_3/2_ → ^4^I_15/2_ (550 nm), ^4^F_9/2_ → ^4^I_15/2_ (670 nm), ^4^S_3/2_ → ^4^I_13/2_ (850 nm) and ^4^I_11/2_ → ^4^I_15/2_ (980 nm). As expected, more discrepancies can be observed for PL of the Er^3+^‐Mn^2+^ co‐doped samples (b and c), where the relative contribution of the particular emissions (Mn^2+^ and/or Er^3+^) in the whole spectrum depends mainly on the effective excitation of the given ion (its absorption characteristics and the λ_ex_ used). Whereas, in the case of the ML effect, the impact of sound/force triggers the overall excitation of the piezoelectric particles, mainly through the conduction band (CB), governed by the triboelectrification and piezoelectrification mechanisms (see Figure 2d), where the emission, i.e., ML, depends on the efficiency of the electron‐hole recombination. In particular, the deep‐trapped electrons stored in the oxygen vacancies and trap levels of Mn^2+^/Er^3+^ are released when subjected to stress stimulation, i.e., acoustic or mechanical waves. The insets given in the discussed spectra (and the ML images in Figure 2g) present photographs of the PL, sound‐induced ML (ML‐US) and friction‐induced ML (ML‐F) of the materials, indicating, in this case, smaller importance of the type of excitation source (i.e. light, sound, or impact), in contrast to the huge influence of the material composition, as discussed in the previous paragraph. Hence, in this particular case, using 375 nm UV laser seems to be a good option for comparing PL and ML phenomena in the material studied. It is worth noting, that the relative ML intensity of Er^3+^, compared to the ML of Mn^2+^ (b and c), is higher for the sound‐induced ML (center) than for the friction‐induced ML (bottom), indicating that the type of mechanical stimuli affects in some extent the energy migration mechanisms and the resulting ML characteristics in such complex systems. The calculated color coordinates for the recorded PL and ML spectra, compared in the CIE diagram in Figure 2e, align with the above‐discussed emission color changes observed in the photographs.

The inset plots in the bottom row of Figure 2a–c illustrate the dependence of ML intensity on the applied mechanical force calculated by integrating ML spectra over the wavelength (in the whole measured wavelength range) and over time. Initially, the mechanoluminescence intensity rapidly increases with force, displaying a steep growth between ≈2 and 15 N. Beyond this region, the intensity increment gradually diminishes, showing a slower growth rate, and approaches saturation at forces ≈25–30 N. This behavior indicates a nonlinear relationship, suggesting that the luminescent centers responsible for mechanoluminescence become progressively saturated or fully activated at higher mechanical forces, especially considering the contribution of different mechanisms, i.e. triboelectrification and piezoelectrification, responsible for the ML phenomena. Nonetheless, no significant changes were observed in the shape of the luminescence spectra of the studied materials with increasing force. The force‐dependent ML spectra and additional information regarding the stability of the friction‐induced ML, together with the related discussion, are provided in the Supporting Information file (Figures S4 and S5, Supporting Information).

Additionally, we were able to record the low‐energy NIR emissions of Er^3+^, i.e., ^4^I_13/2_ → ^4^I_15/2_ transition, centered at ≈1550 nm, both for PL and ML excitation modes, confirming excellent luminescence performance of the materials studies (see Figure 2f). In this case, for technical reasons, we used only focused ultra‐sound to induce the ML phenomenon. Such NIR emission might be significant from the point of view of bioapplications, due to its high permeability through biological systems, i.e., tissues,^[^ ^27^, ^44^, ^45^ ^]^ opening new horizons in non‐invasive optical sensing and imaging (even without excitation light, in the case of sound‐induced ML). We also recorded videos visualizing the phenomena of force‐ and sound‐to‐light conversions for the materials studied (Videos S1–S3, Supporting Information data).

Finally, we used the developed materials for handwriting visualization, to show their high susceptibility to even low frictional force applied and display their friction‐induced ML colors. The obtained results (Figure 2g) clearly show the handwritten letters and symbols, visualized by the use of the thin layers of the ML‐active powder materials deposited on the glass plates and covered with a transparent adhesive tape. The fingernail‐induced ML photographs of the investigated materials' ML colors are displayed on the left side in a series, together with the corresponding 2D (center) and 3D (right) images, obtained upon transformation of their intensities into numerical matrices.

### 2.4 Luminescence Thermometry

**Figure 3:**
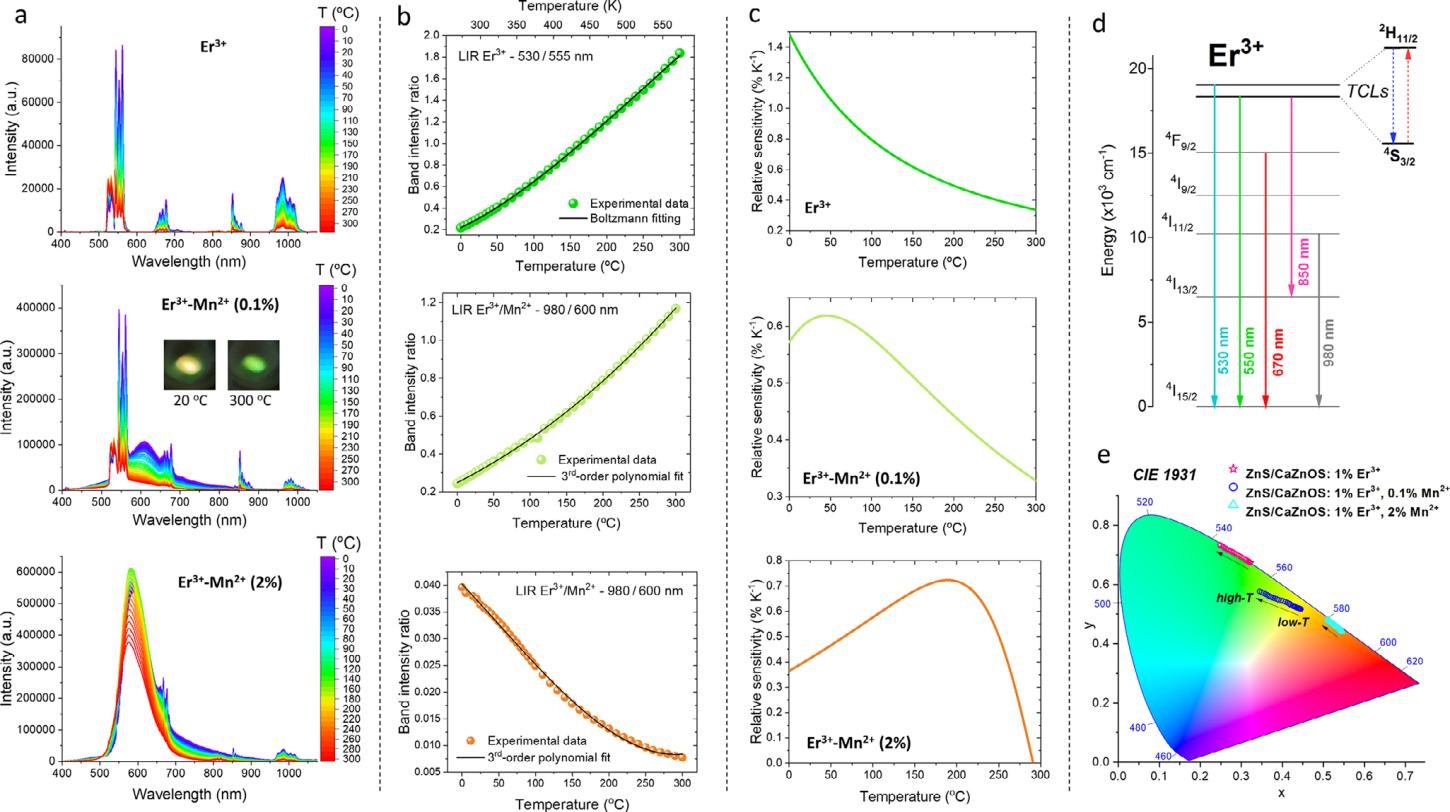
a) Emission spectra for the materials doped with 1% Er^3+^ (top) and co‐doped with 0.1% Mn^2+^ (center) or 2% Mn^2+^ (bottom), at different temperature values, using 375 nm UV laser excitation. b) Determined band intensity ratios, i.e., LIR Er^3+^ 530/555 nm, for the single‐doped sample (top); and LIR Er^3+^/Mn^2+^ 980/600 nm for the samples co‐doped with 0.1% Mn^2+^ (center) and 2% Mn^2+^ (bottom), plotted as a function of temperature. c) The corresponding relative sensitivity values (% K^−1^) as a function of temperature. d) Energy level diagram indicating different radiative transitions of Er^3+^ and emphasizing thermalization of the excited electrons within its TCLs used for Boltzmann‐type luminescence thermometry. e) Chromaticity diagram showing thermal evolution of the color coordinates for the materials studied, based on the corresponding PL spectra.

To extend the functionality and applicability of the materials synthesized, we have investigated their optical response under high‐temperature conditions to use their force‐ and sound‐induced ML activity for remote temperature monitoring in real‐world applications. To do that, first, we have calibrated their PL features with temperature (from 273 to 573 K), taking into account alike Er^3+^ thermally‐coupled levels (TCLs), conforming Boltzmann distribution, as well as the non‐TCLs for Er^3+^/Mn^2+^, as shown in **Figure** **3**. The UV‐excited (375 nm laser) emission spectra, recorded as a function of temperature for all samples, reveal the expected overall decrease in PL intensity with temperature, alike for Er^3+^ and Mn^2+^ emissions (Figure 3a). Nevertheless, the relative intensity of the thermalized band of Er^3+^, centered at ≈530 nm (^2^H_11/2_ → ^4^I_15/2_ transition), increases with temperature due to the thermal population of the excited electrons from the lower‐lying ^4^S_3/2_ state. In other words, ^2^H_11/2_ and ^4^S_3/2_ are TCLs and conform Boltzmann distribution, as depicted in the energy level diagram in Figure 3d. In fact, the luminescence intensity ratio (LIR) of the related Er^3+^ emission bands (530/555 nm) is commonly used in luminescence thermometry applications, and its temperature dependence is well‐established in the literature.^[^ ^25^, ^45^, ^46^ ^]^ The mentioned 530/555 nm LIR parameter, for the Er^3+^ single‐doped sample, as a function of temperature is plotted in Figure 3b (top), resulting in the relative sensitivity of ≈1.3 %/K at room temperature, as shown in Figure 3c (top). Note, the details of luminescence thermometry calculations, including fitting procedure, equation used and parameter values (see Table S1, Supporting Information), Boltzmann relation, LIR and sensitivity calculations, etc., are given in the Supporting Information file.

In the case of the samples co‐doped with Mn^2+^, the mentioned TCLs of Er^3+^ significantly overlap with broad‐band emission of Mn^2+^, hence we were unable to reliably determine thermal evolution of the 530/555 nm LIR parameter. That is why we decided to use determined the LIR between the non‐TCLs of Er^3+^ and Mn^2+^, in the spectra ranges free from the band overlapping, i.e. I) the whole band centered ≈980 nm, associated with the ^4^I_11/2_ → ^4^I_15/2_ transition of Er^3+^; and II) the band of Mn^2+^ integrated in the range from 580 to 630 nm. The resulting 980/600 nm (Er^3+^/Mn^2+^) LIR parameter plotted as a function of temperature increases for the sample with 0.1% of Mn^2+^ (Figure 3b; center), but decreases for the sample with 2% of Mn^2+^ with temperature elevation (bottom). Note that in both cases, the intensity of Er^3+^ emission decreases with temperature at a similar rate, whereas the intensity of Mn^2+^ emission diminishes much faster for the sample with a low concentration of Mn^2+^, compared to the highly‐doped material. Such behavior may be associated with complex character of the energy transfer processes between Er^3+^ and Mn^2+^ ions, including back energy transfer, thermalization of states, energy migration, as well as temperature‐dependent cross‐relaxation processes. Moreover, plausibly with increasing doping concentration the sites available for Mn^2+^ ions become occupied non‐uniformly, and the site with preferential occupation in the lightly‐doped sample in more susceptible for thermal quenching.

Due to the overall complexity of mentioned processes, their interdependences and mutual contribution, we could not use any well‐established physical model to correlate the observed changes of the LIR parameter with temperature. That is why, to correlate their thermal evolutions we applied a simple empirical model, i.e. 3^rd^‐order polynomial fit, as commonly used in such situation, where the non‐TCLs of a single or multi‐ions are utilized for ratiometric luminescence thermometry. The calculated sensitivities plotted in Figure 3c (Mn^2+^ 0.1%‐center; 2%‐bottom) indicate the maximum sensitivity exceeding 0.6 %/K ≈50 °C for the sampled with 0.1% of Mn^2+^, and above 0.7%/K ≈200 °C for the sampled with 2% of Mn^2+^. Interestingly, in the case of the material with 1% of Er^3+^ and 0.1% of Mn^2+^, where their emissions were at similar intensity level, the resulting luminescence color significantly changed with from yellow to green, by elevating temperature from RT to 300 °C. Such behavior could be useful in colorimetric, visual luminescence thermometry applications. These observations were quantified by plotting the temperature‐dependent color coordinates in the CIE diagram displayed in Figure 3e. Note, that in order to improve the thermal sensitivity of the developed ML‐based sensor materials one could use e.g., I) different host matrix; II) play with the doping concentration; III) co‐dope materials with other d‐block metal ions; and IV) use other lanthanides with larger energy separation (ΔE), such as Tm^3+^.

### 2.5 Combination of Sound‐Induced ML with Luminescence Thermometry

**Figure 4:**
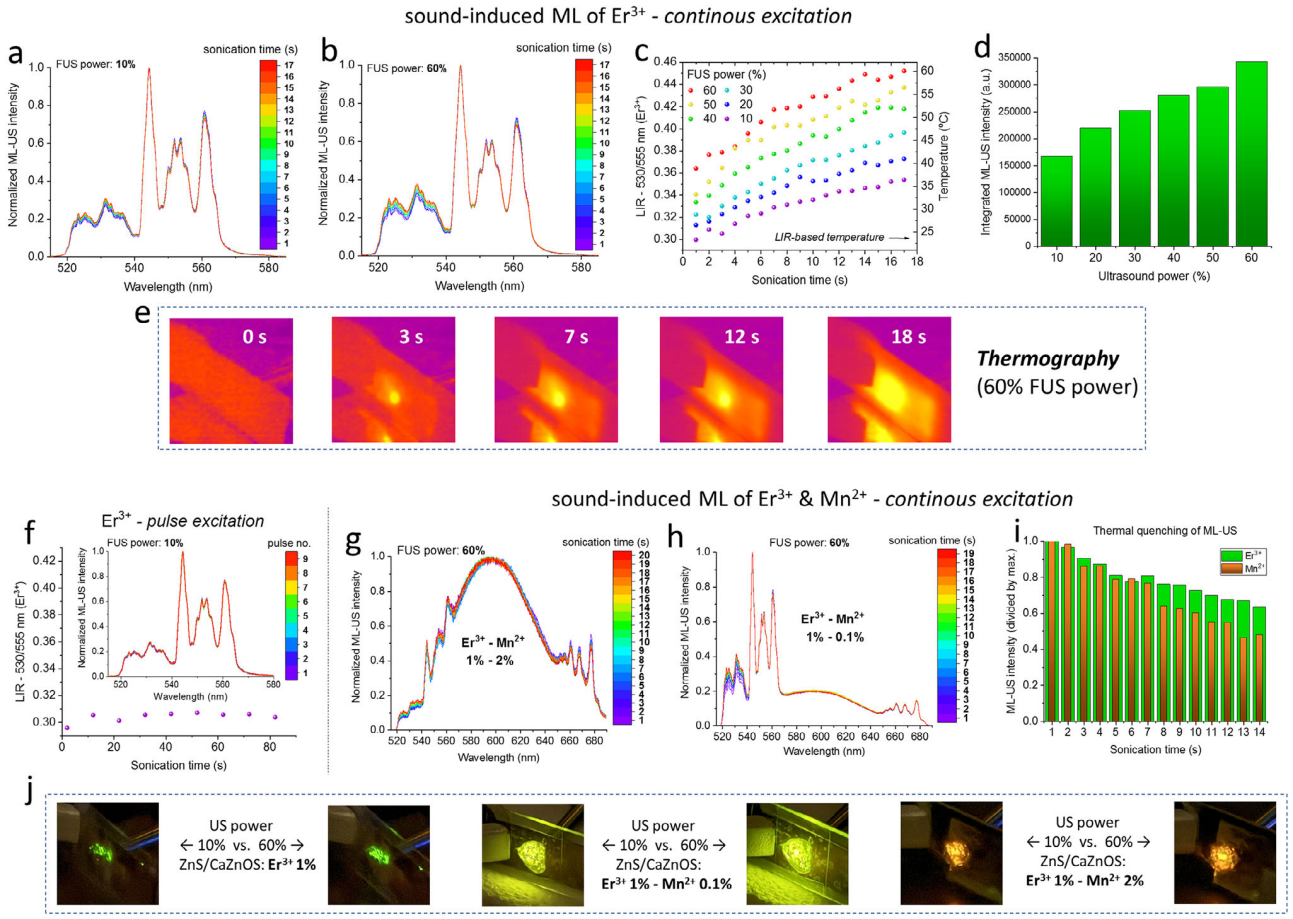
a,b) Sound‐induced ML spectra for the Er^3+^ single‐doped material, recorded at 10% and 60% of FUS power, at different sonication times. c) The determined 530/555 nm LIR values determined for different FUS power (10–60%) plotted as a function of sonication time; the right y‐axis shows the corresponding temperature values, calculated based on the calibration from luminescence thermometry, i.e. relation of LIR versus temperature given in Figure 3b (top). d) Integrated sound‐induced ML intensity as a function of FUS power. e) Representative thermographic images for the Er^3+^‐doped sample exposed to 60% of FUS (continuous sonication), taken at different time intervals. f) Sound‐induced ML spectra for the Er^3+^‐doped sample, recorded during pulsed excitation (FUS power of 10%; pulse duration: 1 s; frequency: 0.1 Hz), displayed together with the corresponding 530/555 nm LIR values plotted as a function of sonication time (pulse number). g,h) Sound‐induced ML spectra for the samples co‐doped with 1% Er^3+^ and 2% Mn^2+^ or 0.1% Mn^2+^, recorded at 60% of the FUS power, with increasing time of continuous sonication. i) Integrated sound‐induced ML intensity (divided by a maximum value) as a function of sonication time (FUS power of 60%) for the Er^3+^‐Mn^2+^ co‐doped material, indicating different thermal quenching rates for both ions. j) Photographs of the samples' multi‐color ML triggered by sonication with the FUS source of different powers (10 and 60%).

In order to study in detail, the sound‐to‐light conversion and the accompanied heat generation, monitored by the use of luminescence thermometry, first we used the Er^3+^ single‐doped material. The sample powder was placed on the glass plate forming a uniform layer (≈100 µm thick), then it was covered and glued with a standard, transparent adhesive tape (from the detection side). Whereas from the back side we attached a piece of copper sheet (≈200 µm thick; 2 × 2 cm), which directly touched the flat metal tip of the focused ultrasound (FUS) device, in order to dissipate the excess of heat, to avoid overheating of the system. **Figure** **4**a,b show the normalized ultrasound‐induced (US‐ML) spectra, acquired at 10% and 60% of the FUS power as a function of time (continuous US‐excitation), respectively, in the range of 515–580 nm, centered to the green emission of Er^3+^. It is clear, that together with increased FUS power and sonication time the local temperature elevation of the ML‐active material occurs (acoustic heating), manifested as increased relative intensity of the high‐energy band of Er^3+^ (at 530 nm), leading to the increase of the 530/555 nm LIR parameter. This is due to the occurrence of thermalization of Er^3+^ states, caused by the sound‐to‐heat conversion (accompanied with the sound‐to‐light conversion–ML). This is quantitatively shown in Figure 4c, where the determined LIR values as a function of time are plotted for six values of the FUS power (10, 20, 30, 40, 50, and 60%). The right Y‐axis represents the calculated local temperature values based on the LIR of TCLs of Er^3+^ and the performed calibration curve, i.e. correlation of LIR with temperature given in Figure 3b (top). The obtained *T*‐values indicate the increase of the local temperature from RT up to ≈60 °C. Additionally, we showed in Figure 4d the dependence of the total ML intensity as a function of FUS power, indicating a clear increasing tendency. Whereas the representative thermographic images of the heat dissipation for the continuous sonication of the sample at 60% of FUS, for different time intervals, are displayed in Figure 4e. The tendency of increased local temperature, at the contact point of the FUS with the sample recorded with a thermal camera, agrees well with the spectroscopic data from luminescence thermometry, confirming the utility of this method.

While the above results are interesting from the perspective of remote monitoring of the sound‐to‐heat conversion, that approach hampers optical temperature probing of the external systems and devices, due to the inherent intrinsic heat generation during the measurement. To overcome this limitation, and show the full potential of the sound‐induced ML for diverse temperature sensing application, we utilized a pulsed mode of the FUS device, so as to generate relatively short pulses of 1 s duration, with frequency (repetition rate) of 0.1 Hz, i.e. 1 pulse per 10 s (FUS power of 10%). The recorded US‐ML spectra for the Er^3+^‐doped sample shown in Figure 4f, together with the corresponding 530/555 nm LIR values plotted as a function of the sonication time (pulse number), clearly confirm a negligible heating effect and constant local temperature of the sample during the pulsed sonication (thanks to the heat dissipation into the surroundings), allowing reliable temperature probing of the heated systems, which will be utilized and discussed in detail in the next section. Note, that in order to minimize the US‐induced heating effects one can use: I) shorter US pulses; II) decrease the repetition rate; III) use low FUS power; IV) attach a heat sink, such as a metal plate, between the sample and the FUS device to improve heat dissipation.

For the sake of complete analysis, we also investigated the effect of continuous sonication (FUS power of 60%) of the Er^3+^‐Mn^2+^ co‐doped materials, as shown in Figure 4g,h, for the samples with 2% and 0.1% of Mn^2+^, respectively. As expected, the recorded, normalized ML‐US spectra clearly indicate local samples heating, manifested in the increased 530/555 nm LIR values with sonication time, due to the accompanying heat generation resulting in thermalization of Er^3+^ TCLs. However, as we disuse din the previous section, due to the significant Er^3+^ and Mn^2+^ bands overlapping, as well as the complex temperature influence on the ML mechanisms for both ions and their mutual interdependences, we were unable to unambiguously quantify the corresponding LIR values and reliably determine the local temperature gradient, by the use of ML of the co‐coped materials and luminescence thermometry approach. Note, that for the continuous sonication, due to the technical reasons the spectra had to be recorded in a kinetic mode, limiting to the acquired spectral range to the single frame which can be recorded with our CCD camera, hampering simultaneous acquisition of the Er^3+^ band at 980 nm, which was previously used for the temperature calibration via PL‐based ratiometric thermometry, in the case of the co‐doped samples. Additionally, in Figure 4i the evolutions of the ML‐US intensities for both Er^3+^ and Mn^2+^ ions are plotted as a function of sonication time, revealing faster thermal quenching rate of the sound‐induced ML for the Mn^2+^ than for Er^3+^. This is plausibly associated with different ML mechanisms and temperature dependences for both ions, as has been already mentioned. Finally, to better visualize the impact of sonication power and the composition of the ML‐active materials on the emitting ML‐US color, the digital photographs of all materials exposed to FUS source of different power (10 and 60%), are displayed in Figure 4j.

### 2.6 Temperature Probing with Sound‐Induced ML of Er^3+^

**Figure 5:**
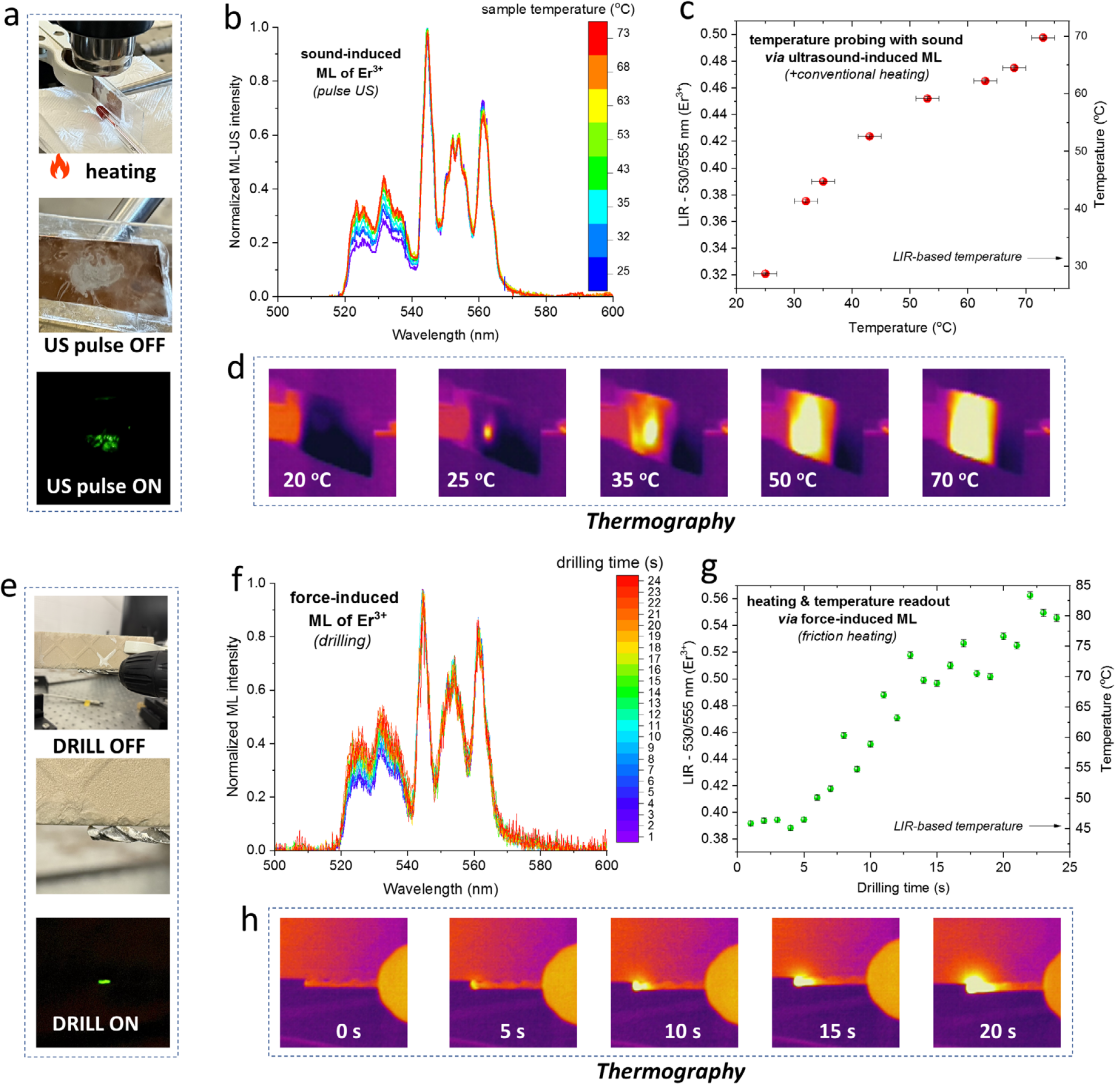
a–d) Results from remote temperature probing using the Er^3+^ single‐doped material with an externally‐heated system. Photographs showing the setup to induce ML with FUS, for the material heated with a heating gun and temperature monitored with an external thermometer (a). Sound‐induced ML spectra captured at different temperature values (b). The determined 530/555 nm LIR values plotted as a function of the adjusted temperature, where the right y‐axis indicates the corresponding temperature valued based on LIR versus *T* calibration (c). Thermographic images visualizing the temperature gradient of the material during external heating (d). e–h) Results from locally‐heated items via mechanical friction (drilling), simultaneously inducing ML of the Er^3+^ single‐doped material, applied for luminescence thermometry. Photographs illustrating the drilling in a ceramic tile, accompanied with green ML, using a drill containing the ML‐active sample (e). Friction‐induced ML spectra recorded during the drilling process (f). The determined 530/555 nm LIR values as a function of drilling time, transformed into temperature values (right y‐axis) via luminescence thermometry (g). Thermographic images displaying the local temperature gradient throughout the drilling process (h). The error bars represented the standard deviations of the data sets.

In this section, we describe the results of remote temperature probing with Er^3+^ single‐doped material of the I) externally‐heated system (**Figure** **5**a–d); and II) locally‐heated items via mechanical friction, i.e., drilling (Figure 5e–h). In the first case, we used the FUS device directed at the ML‐active material placed between the glass and metal plates (as described in the previous section), heating gun, conventional thermometer and thermal camera, in order to induce the ML, increase temperature of the system (by external heating) up to ≈70 °C, and monitor its overall temperature, as can be seen in the photographs displayed in Figure 5a. By setting the heating gun to the given temperature and after reaching the desired value, the generated US pulse induced green ML of Er^3+^, whose light was captured by the optical detection system, and presented as a set of emission spectra in Figure 5b. With temperature elevation, the intensity of the high‐energy band originating from TCL of Er^3+^ at 530 nm gradually increased, leading to the increase of the 530/555 nm LIR plotted as a function of temperature in Figure 5c. It is clear, that the adjusted temperature values (*x*‐axis) correlated well with the thermal readouts from luminescence thermometry (right *y*‐axis). To better visualize the local temperature gradient, the representative thermographic images are shown in Figure 5d.

In the second case, we tightly attached a fine layer of the ML‐active sample (using cyanoacrylate glue) to the surface of the metal drill fixed in a cordless drill, and continuously drill over 20 s onto the surface of the ceramic tile, as presented in the photographs shown in Figure 5e. By starting the drilling process, metal drill rubs against the tile causing friction and generating heat. In the ML‐US emission spectra, recorded during the drilling process and shown in Figure 5f, the thermalization of Er^3+^ bands in clearly observed with drilling time. The determined 530/555 nm LIR values as a function of drilling time (Figure 5g) were transformed into local temperature values, using the established luminescence thermometry calibration, resulting in the local temperature elevation over 80 °C. Again, the selected thermographic images displayed in Figure 5h allow clear visualization of the local temperature gradient in the inspected system during the drilling process. The results presented in this section clearly confirm a great utility and application potential of the force‐ and sound‐induced ML combined with luminescence thermometry of lanthanides, for the excitation‐light free, new generation remote temperature monitoring. It is wort noting, that luminescence thermometry offers improved performance over thermography, as it is not influenced by unknown surface emissivity or background thermal radiation, which hamper and bias temperature readouts with commercially available thermal cameras.^[^ ^46^, ^47^ ^]^

## 3 Conclusion

Here we have shown a successful demonstration of the development of multifunctional, ML‐active materials based on Er^3+^ and Mn^2+^ co‐doped ZnS/CaZnOS heterostructures for remote temperature detection, visual sensing of force and sound, as well as handwriting visualization. The fundamental optical properties of the material were thoroughly investigated using photoluminescence (PL) spectroscopy. Excitation with both ultraviolet (UV) and near‐infrared (NIR) light resulted in characteristic down‐shifting and up‐converting emissions, demonstrating multi‐color emissions depending on the excitation wavelength used and materials composition. The relationship between the applied force or ultrasounds power versus ML intensity was quantitatively analyzed, revealing efficient force‐ and sound‐to‐light conversions in the materials studied, accompanied with additional heat generation, while strong frictional stimuli or continuous sonication with FUS device were applied. Furthermore, the temperature‐dependent PL properties were exploited to establish a reliable luminescence thermometry calibration. By analyzing the emission intensity ratios of thermally‐coupled levels within the Er^3^⁺ ions and non‐thermally‐coupled levels of the Er^3^⁺/Mn^2^⁺ system, we were able to develop accurate temperature sensing platform. The practical utility of these multifunctional materials was demonstrated through a series of experiments involving friction‐ and sound‐induced ML thermometry. We have successfully monitored temperature changes during a drilling process, by detecting the ML generated by mechanical waves. On the other hand, by using pulsed sonication we could remotely monitor the temperature of externally heated systems. Additionally, the materials studied exhibit sound‐to‐heat conversion, enabling continuous temperature elevation (acoustic heating) and its monitoring under sustained sonication. This work highlights the immense potential of integrating sound‐to‐light conversion with luminescence thermometry for remote monitoring applications. The ability to perform excitation‐light‐free luminescence temperature probing in various systems and working devices opens up new avenues for non‐invasive sensing in diverse fields, including manufacturing, medical diagnostics, and environmental monitoring. Moreover, the fascinating and underexplored phenomenon of sound‐to‐light conversion via ML opens new horizons in facile acoustic waves visualization.

## 4 Experimental Section

Detailed experimental procedures are reported in the Supporting Information.

## Conflict of Interest

The authors declare no conflict of interest.

## Supporting information



Supporting Information

Supplemental Video 1

Supplemental Video 2

Supplemental Video 3

## Data Availability

The data that support the findings of this study are available from the corresponding author upon reasonable request.

## References

[1] Z. Xie , Y. Xue , X. Zhang , J. Chen , Z. Lin , B. Liu , Nat. Commun. 2024, 15, 3668. 38693122 10.1038/s41467-024-47962-6 PMC11063035

[2] S. Jiang , X. Wu , F. Yang , N. J. Rommelfanger , G. Hong , Nat. Protoc. 2023, 18, 3787. 37914782 10.1038/s41596-023-00895-8 PMC11405139

[3] B. P. Chandra , A. S. Rathore , Cryst. Res. Technol. 1995, 30, 885.

[4] S. Chang , K. Zhang , D. Peng , Y. Deng , C.‐X. Shan , L. Dong , Nano Energy 2024, 122, 109325.

[5] Y. Zhuang , R. Xie , Adv. Mater. 2021, 33, 2005925. 10.1002/adma.202005925 33786872

[6] W. Li , Y. Cai , J. Chang , J. Liu , S. Wang , J. Zhang , Adv. Funct. Mater. 2025, 35, 2412494.

[7] C. D. S. Brites , X. Xie , M. L. Debasu , X. Qin , R. Chen , W. Huang , J. Rocha , X. Liu , L. D. Carlos , Nat. Nanotechnol. 2016, 11, 851. 27376242 10.1038/nnano.2016.111

[8] S. Chang , K. Zhang , D. Peng , Y. Deng , C.‐X. Shan , L. Dong , Nano Energy 2024, 122, 109325.

[9] J. R. Casar , C. A. McLellan , C. Shi , A. Stiber , A. Lay , C. Siefe , A. Parakh , M. Gaerlan , X. W. Gu , M. B. Goodman , J. A. Dionne , Nature 2025, 637, 76. 39743609 10.1038/s41586-024-08331-x PMC12755916

[10] Y. Bai , X. Guo , B. Tian , Y. Liang , D. Peng , Z. Wang , Adv. Sci. 2022, 9, 2203249. 10.1002/advs.202203249 PMC9534939 35975462

[11] Z. Huang , B. Chen , B. Ren , D. Tu , Z. Wang , C. Wang , Y. Zheng , X. Li , D. Wang , Z. Ren , S. Qu , Z. Chen , C. Xu , Y. Fu , D. Peng , Adv. Sci. 2023, 10, 2204925. 10.1002/advs.202204925 PMC9875687 36372543

[12] X. Cao , H. Yang , Z.‐L. Wu , B.‐B. Li , Light Sci. Appl. 2024, 13, 159. 38982066 10.1038/s41377-024-01480-8 PMC11233744

[13] T. G. Leighton , Prog. Biophys. Mol. Biol. 2007, 93, 3. 17045633 10.1016/j.pbiomolbio.2006.07.026

[14] D. Le , D. Dhamecha , A. Gonsalves , J. U. Menon , Front. Bioeng. Biotechnol. 2020, 8, 25. 32117914 10.3389/fbioe.2020.00025 PMC7016203

[15] Q. Zhang , M. Xu , L. Zhou , S. Liu , W. Wang , L. Zhang , W. Xie , C. Yu , Nat. Commun. 2023, 14, 1257. 36878901 10.1038/s41467-023-36916-z PMC9988937

[16] H. G. Shin , S. Timilsina , K. Sohn , J. S. Kim , Adv. Sci. 2022, 9, 2105889. 10.1002/advs.202105889 PMC9008412 35156335

[17] F. Yang , H. Cui , X. Wu , S.‐J. Kim , G. Hong , Nanoscale 2023, 15, 1629. 36625323 10.1039/d2nr06129e PMC10505055

[18] Y. Ding , B. So , J. Cao , L. Wondraczek , Adv. Sci. 2022, 9, 2201631. 10.1002/advs.202201631 PMC9376836 35712779

[19] K. Chang , J. Gu , L. Yuan , J. Guo , X. Wu , Y. Fan , Q. Liao , G. Ye , Q. Li , Z. Li , Adv. Mater. 2024, 36, 2407875. 10.1002/adma.202407875 39049679

[20] W. Wang , A. Tasset , I. Pyatnitskiy , H. G. Mohamed , R. Taniguchi , R. Zhou , M. Rana , P. Lin , S. L. C. Capocyan , A. Bellamkonda , W. Chase Sanders , H. Wang , Adv. Drug Delivery Rev. 2022, 186, 114343. 10.1016/j.addr.2022.114343 PMC10202817 35580814

[21] Y. Ding , B. So , J. Cao , F. Langenhorst , L. Wondraczek , Adv. Opt. Mater. 2023, 11, 2300331.

[22] T. Zheng , M. Runowski , N. Stopikowska , M. Skwierczyńska , S. Lis , P. Du , L. Luo , J. Alloys Compd. 2022, 890, 161830.

[23] T. Zheng , L. Zhou , X. Qiu , D. Yang , M. Runowski , S. Lis , P. Du , L. Luo , J. Lumin. 2020, 227, 117517.

[24] P. Woźny , K. Soler‐Carracedo , M. Perzanowski , J. Moszczyński , S. Lis , M. Runowski , J. Mater. Chem. C 2024, 12, 11824.

[25] M. Runowski , P. Woźny , S. Lis , V. Lavín , I. R. Martín , Adv. Mater. Technol. 2020, 5, 1901091.

[26] S. Goderski , M. Runowski , P. Woźny , V. Lavín , S. Lis , ACS Appl. Mater. Interfaces 2020, 12, 40475. 32805851 10.1021/acsami.0c09882 PMC7498144

[27] E. C. Ximendes , U. Rocha , T. O. Sales , N. Fernández , F. Sanz‐Rodríguez , I. R. Martín , C. Jacinto , D. Jaque , Adv. Funct. Mater. 2017, 27, 1702249.

[28] A. Skripka , A. Benayas , R. Marin , P. Canton , E. Hemmer , F. Vetrone , Nanoscale 2017, 9, 3079. 28252155 10.1039/c6nr08472a

[29] Y. Shen , L. Liang , S. Zhang , D. Huang , J. Zhang , S. Xu , C. Liang , W. Xu , Nanoscale 2018, 10, 1622. 29239454 10.1039/c7nr08636a

[30] C. D. S. Brites , S. Balabhadra , L. D. Carlos , Adv. Opt. Mater. 2019, 7, 1801239.

[31] J. Zhou , B. Del Rosal , D. Jaque , S. Uchiyama , D. Jin , Nat. Methods 2020, 17, 967. 32989319 10.1038/s41592-020-0957-y

[32] C. Li , Q. He , Y. Wang , Z. Wang , Z. Wang , R. Annapooranan , M. I. Latz , S. Cai , Nat. Commun. 2022, 13, 3914. 35798737 10.1038/s41467-022-31705-6 PMC9263131

[33] X. Pan , Y. Zhuang , W. He , C. Lin , L. Mei , C. Chen , H. Xue , Z. Sun , C. Wang , D. Peng , Y. Zheng , C. Pan , L. Wang , R.‐J. Xie , Nat. Commun. 2024, 15, 2673. 38531867 10.1038/s41467-024-46900-w PMC10966096

[34] Y. Zhao , G. Bai , Y. Huang , Y. Liu , D. Peng , L. Chen , S. Xu , Nano Energy 2021, 87, 106177.

[35] R. R. Petit , S. E. Michels , A. Feng , P. F. Smet , Light Sci. Appl. 2019, 8, 124. 31885866 10.1038/s41377-019-0235-x PMC6930285

[36] L. Liu , C. Xu , A. Yoshida , D. Tu , N. Ueno , S. Kainuma , Adv. Mater. Technol. 2019, 4, 1800336.

[37] D. Tu , C. Xu , A. Yoshida , M. Fujihala , J. Hirotsu , X. Zheng , Adv. Mater. 2017, 29, 1606914. 10.1002/adma.201606914 28370452

[38] X. Qiu , J. Liu , B. Zhou , X. Zhang , Adv. Funct. Mater. 2023, 33, 2300321.

[39] Z. Ma , J. Zhou , J. Zhang , S. Zeng , H. Zhou , A. T. Smith , W. Wang , L. Sun , Z. Wang , Mater. Horiz. 2019, 6, 2003.

[40] Y. Zhao , D. Peng , G. Bai , Y. Huang , S. Xu , J. Hao , Adv. Funct. Mater. 2021, 31, 2010265.

[41] D. Peng , Y. Jiang , B. Huang , Y. Du , J. Zhao , X. Zhang , R. Ma , S. Golovynskyi , B. Chen , F. Wang , Adv. Mater. 2020, 32, 1907747. 10.1002/adma.201907747 32128925

[42] T. Zheng , M. Runowski , I. R. Martín , K. Soler‐Carracedo , L. Peng , M. Skwierczyńska , M. Sójka , J. Barzowska , S. Mahlik , H. Hemmerich , F. Rivera‐López , P. Kulpiński , V. Lavín , D. Alonso , D. Peng , Adv. Mater. 2023, 35, 2304140. 10.1002/adma.202304140 37399662

[43] Y. Du , Y. Jiang , T. Sun , J. Zhao , B. Huang , D. Peng , F. Wang , Adv. Mater. 2019, 31, 1807062. 10.1002/adma.201807062 30589165

[44] N. Jurga , M. Runowski , T. Grzyb , J. Mater. Chem. C 2024, 12, 12218.

[45] C. D. S. Brites , A. Millán , L. D. Carlos , in Handb. Phys. Chem. Rare Earths, Elsevier, Amsterdam, Netherlands, 2016, pp. 339–427.

[46] C. D. S. Brites , R. Marin , M. Suta , A. N. Carneiro Neto , E. Ximendes , D. Jaque , L. D. Carlos , Adv. Mater. 2023, 35, 2302749. 10.1002/adma.202302749 37480170

[47] C. Hernández‐Álvarez , P. I. Martín‐Hernández , I. R. Martín , F. Rivera‐López , H. Hemmerich , M. Grzegorczyk , S. Mahlik , M. Runowski , Adv. Opt. Mater. 2024, 12, 2303328.

