# Supplementary material for: Sound, Force and Light Induced Emissions from Er3+‐Mn2+ Doped ZnS/CaZnOS Heterostructure for Remote Temperature Monitoring via Photo‐ and Mechanoluminescence
Source: Adv Mater. 2025 Jul 23;37(41):e10117. doi: 10.1002/adma.202510117 (PMC12531744; doi:10.1002/adma.202510117)
Supplement: Supplementary file 1 — Supporting Information [file ADMA-37-e10117-s002.docx]

Supporting Information for:

**Sound, Force and Light Induced Emissions from Er^3+^-Mn^2+^ doped ZnS/CaZnOS Heterostructure for Remote Temperature Monitoring via Photo- and Mechanoluminescence**

Marcin Runowski,* Jan Moszczyński, Przemysław Woźny, Kevin Soler-Carracedo, Justyna Barzowska, Sebastian Mahlik, Dengfeng Peng*, Teng Zheng*

Prof. M. Runowski, Jan Moszczyński, Dr. Przemysław Woźny, Dr. K. Soler-Carracedo

Faculty of Chemistry

Adam Mickiewicz University

Poznań 61-614, Poland

E-mail: runowski@amu.edu.pl

Dr. T. Zheng, Prof. L. Peng

School of Information and Electrical Engineering,

Hangzhou City University

Hangzhou 310015, China

E-mail: zhengteng@zucc.edu.cn

Dr. J. Barzowska, Prof. S. Mahlik

Institute of Experimental Physics

Faculty of Mathematics, Physics and Informatics,

University of Gdansk,

Gdansk 80-308, Poland

Prof. D. Peng

Shenzhen Key Laboratory of Intelligent Optical Measurement and Detection, ShenzhenUniversity, Shenzhen 518060, China;

Key Laboratory of Optoelectronic Devices and Systems of Ministry of Education andGuangdong Province, College of Physics and Optoelectronic Engineering, ShenzhenUniversity, Shenzhen 518060, China;

State Key Laboratory of Radio Frequency Heterogeneous Integration, Shenzhen University. Shenzhen 518060, China

E-mail: pengdengfeng@szu.edu.cn

**Experimental section**

**1. Synthesis of the ZnS/CaZnOS:Er^3+^ and ZnS/CaZnOS:Er^3+^-Mn^2+^ materials**

The following protocol refers to the optimal synthesis conditions of the Er^3+^-doped, and Er^3+^-Mn^2+^ co-doped ZnS/CaZnOS heterostructure materials, synthesized *via* solid state method. When doping the CaZnOS compound, the Mn^2+^ dopant ions replace the Zn^2+^ host ions, and the Er^3+^ dopant ions replace the Ca^2+^ host ions.

I) To obtain the ZnS/CaZnOS doped with 1 mol% of Er^3+^, the appropriate amounts of raw solid materials were used as starting materials: ZnS (1.41618 g, 14.531 mmol, 99.99%, Sigma Aldrich), CaCO_3_ (0.57594g, 5.754 mmol, 99.5%, Acros Organics), Er_2_O_3­_ (0.01112 g, 0.029 mmol, 99.99%, Sigma Aldrich) and LiNO_3_ (0.02404 g, 0.349 mmol, 99%, Alfa Aesar).

II) To obtain the ZnS/CaZnOS co-doped with 1 mol% of Er^3+^ and 1 mol% of Mn^2+^, the following amounts of raw materials were used: ZnS (1.41562 g, 14.525 mmol, 99.99%, Sigma Aldrich), CaCO_3_ (0.57594g, 5.754 mmol, 99.5%, Acros Organics), MnCO_3_ (0.00067 g, 0.006 mmol, >99.9%, Sigma Aldrich), Er_2_O_3­_ (0.01112 g, 0.029 mmol, 99,99%, Sigma Aldrich) and LiNO_3_ (0.02404 g, 0.349 mmol, 99%, Alfa Aesar).

III) To obtain the ZnS/CaZnOS co-doped with 1 mol% of Er^3+^ and 2 mol% of Mn^2+^, the following amounts of raw materials were used: ZnS (1.40485 g, 14.415 mmol, 99.99%, Sigma Aldrich), CaCO_3_ (0.57594 g, 5.754 mmol, 99.5%, Acros Organics), MnCO_3_ (0.01336 g, 0.116 mmol, >99.9%, Sigma Aldrich), Er_2_O_3­_ (0.01112 g, 0.029 mmol, 99.99%, Sigma Aldrich) and LiNO_3_ (0.02404g, 0.349 mmol, 99%, Alfa Aesar). In all cases LiNO_3_ was used as flux compound, and was calculated, as the rest of precursors, for 2g of final heterostructure compound. All precursors were mixed and grounded homogeneously in separate, cleared earlier, agate mortar with ethanol as grinding medium. After grinding mixtures was dried in oven at 80^o^C to eliminate rests of ethanol in grinding mixture. After that obtained powder was calcinated in tubular furnace at temperature of 1050^o^C for 4h under protective atmosphere of Ar (≥99,999%, Linde) in quartz crucible. Final products were naturally cooled to room temperature and then grounded in clean agate mortar again for further detailed characterization.

**2. Characterization**

*Basic characteristics*

X-ray diffraction patterns (XRD) were measured with a Bruker AXS D8 Advance diffractometer (6°-60° 2θ range) using Cu Kα1 radiation (λ=1.5406 Å). Absorption spectra were acquired in diffused-reflection mode, using a JASCO V-770 UV-Vis/NIR spectrophotometer, equipped with an ILN-925 spherical integrator (150 mm in diameter). Scanning electron microscopy (SEM) images were taken with a FEI Quanta 250 FEG, equipped with an EDAX detector. The Raman spectra were recorded in backscattering geometry with a Renishaw InVia confocal micro-Raman system, using a grating with 1200 grooves/mm and a power-controlled 532 nm laser diode. Excitation and emission spectra (UV-excited) were recorded using a Hitachi F-7000 spectroﬂuorometer equipped with a 150 W xenon lamp, and they were corrected for the apparatus response. The up-conversion emission spectra (NIR-excited) were measured with an Andor Shamrock 500i spectrometer, coupled with a silicon CCD camera (iDus), and a 975 nm NIR continuous wave laser from CNI, with a maximum output power of 2 W, as an excitation source.

*Friction-induced ML*

A home-built setup was used to measure the mechanical friction-induced ML. The mechanical stimulation was performed automatically using a system controlled by dedicated custom-made software in the LabView environment. The powdered samples were spread on the PMMA plate for the ML experiments, forming a layer about 50 μm thick. The glass rod mounted on the linear rail was dragged over the plate with the sample. The rod was pressed toward the sample with the setting force, and a set number of movements were performed at the set speed and frequency. The ML signal was collected using a Shamrock 500i spectrometer with a CCD camera iDus420 (Andor Technology). The glass rod was pressed against the sample plate with a defined load ranging from 2 N to 30 N and subsequently dragged repeatedly across the same area at a constant speed of 6 mm/s, maintaining a time interval of 5 s between each pass. The ML spectra were collected every 60 ms.

*Sound-induced ML*

The ultrasounds of frequency 20 kHz ±500 Hz were generated with the ultrasonic homogenizer (focused ultra-sonicator) BANDELIN Sonopuls HD 2070.2, with a maximum output power of 70 W. To record the sound-induced ML spectra, a spectrometer (Andor Shamrock 500i) with a silicon CCD camera (iDus) detector was applied. All luminescence spectra were corrected for the apparatus response of the system.

*Temperature calibration*

The Linkam THMS600 heating-cooling stage temperature controller, coupled with the LNP95 liquid nitrogen cooling pump system, was for temperature-dependent measurements, in the range of 0-300 ^o^C. The temperature-dependent PL spectra were measured with an Andor Shamrock 500i spectrometer, coupled with a silicon CCD camera (iDus), and a 375 nm UV continuous wave laser, with a maximum output power of 100 mW, as an excitation source. The thermal images were taken with a thermographic camera Sonel KT-200.

**3. Statistical Analysis**

The data integration, normalization, derivation, and graph preparation were performed using

Origin 2021 software, Microsoft Excel, PowerPoint and Diamond. The normalization of the

data (PL emission/excitation spectra) were performance by normalizing all spectra/data to the

most intense peaks (maxima = 1). The standard deviations (SD) obtained in the experiments

are corresponded to the error bars.

**Figure S1.** EDX spectra for the synthesized heterostructure materials ZnS/CaZnOS doped with Er^3+^ and co-doped with Er^3+^-Mn^2+^ ions.

**Figure S2.** Non-normalized up-conversion emission spectra (λ_ex_ = 975 nm NIR laser) recorded for the materials studied, doped with Er^3+^ and co-doped with Er^3+^-Mn^2+^ ions.

We have used the well-known relation *I_em_* ∝ (*I*_pump_)*^n^* to determine the number of photons participating in the transitions corresponding to the observed emission bands of Er^3+^ in the materials obtained (see the up-conversion emission spectra in Figures 1k and S2, in the main manuscript and in the supporting information files, respectively). In the mentioned relation, *I_em_* is the emission intensity, *I*_pump_ is the laser power and *n* is the number of photons involved in the given transition. Applying a linear fit, *n* can be extracted from the slopes of the plotted (in logarithmic scales) emission intensity as a function of the pump power. Please note, that this procedure is commonly used for up-conversion (UC) processes. Figure S3 shows the obtained log-log plots of the integrated luminescence intensities of all observed up-conversiion emission bands of Er^3+^ as a function of laser power, for the materials studied. The linear fits were applied in the whole excitation power range used. The determined slope values are given in Figure S3. The slope values for the UC emission bands of Er^3+^ are close to the theoretical value of 2, confirming the expected two-photon UC processes, governing the transitions associated with those bands. The deviations from the ideal values (2), expected for the pure two-photon processes are related to different non-radiative quenching mechanisms of the excited states of Er^3+^, namely multi-phonon relaxation and cross-relaxation processes.

**Figure S3.** Dependences of the integrated green UC-PL intensity *vs*. the excitation power (log-log plot), recorded for the materials studied, doped with Er^3+^ and co-doped with Er^3+^-Mn^2+^ ions (λ_ex_ = 975 nm NIR laser). The slope values extracted from power-law fits (bold lines) correspond to the number of photons participating in the up-conversion photoluminescence of Er^3+^.

**Luminescence Thermometry**

Er^3+^ ions present thermally-coupled levels (TCLs) at around 532 and 550 nm (^2^H_11/2_ → ^4^I_15/2_ and ^4^S_3/2_ → ^4^I_15/2_). TCLs present relative intensity increases between their bands due to thermalization processes. These thermalization processes follow a Boltzmann-type distribution:

$LIR\equiv\frac{I_{2}}{I_{1}}=B\exp\left( -\frac{\Delta E}{k_{B}T} \right)$ (1)

where *LIR* is the luminescence intensity ratio of the higher and lower-energy bands (*I*_2_; 530 nm and *I*_1_; 555 nm, respectively); Δ*E* is the energy difference between the barycenters *I*_2_ and *I*_1_; *k_B_* is the Boltzmann constant; *T* is the absolute temperature; and *B* is a constant, which depends on the rates of total spontaneous emission, states degeneracies, branching ratio of the transitions in respect to the ground state, and transitions angular frequencies ^1–3^. In Figure 4b (top) it can be seen the obtained LIR values and the corresponding fit, resulting in an energy difference of Δ*E = 763.22 cm^-1^* and a constant value of *B = 10.01*.

In the case of using bands related to different ions (In this case 980/600 nm for Er^3+^/Mn^2+^, respectively) to sense changes in temperature by their relative intensity changes, the bands cannot be considered TCLs and, therefore, the previous Boltzmann-type distribution ^4^. These type of optical sensors are categorized as secondary type sensor ^5^. Given the lack of an appropriate physical model, the non-TCLs were fitted to an empirical third-order polynomial function:

$LIR\equiv\frac{I_{2}}{I_{1}}=y_{0}+AT+BT^{2}+CT^{3}$ (2)

where *y0* corresponds to the vertical offset, T to the absolute temperature and A, B, and C are constants. The values obtained from the fittings presented in Figure 4b (center and bottom) are listed in Table S1.

Table S1. LIR fitting values.

| Sample | y_0_ | A | B | C |
| --- | --- | --- | --- | --- |
| Er^3+^-Mn^2+^ (0.1%) | *0.249* | *0.0019* | *3.50E-6* | *8.05E-10* |
| Er^3+^-Mn^2+^ (2%) | *0.402* | *-1.46E-4* | *-1.21E-7* | *8.54E-10* |

In order to investigate the performance of an optical nanothermometer, it is important to determine the parameter known as relative sensitivity (*S_R_*). Relative temperature sensitivity is related to the change of the analyzed thermometric parameter (LIR in this case) with respect to 1 ºC, and it is usually expressed in % ºC^-1^. This parameter allows to compare the performance between different optical sensors, even when they are based in different approaches such as LIR, single intensity, lifetimes, etc. It is described by Eq. 3:

*𝑆_R_* = 100% × $\frac{dLIR}{dT} \frac{1}{LIR}$ (3)

**Friction-induced ML** (quantification and stability)

Figure S4a-e show the results of the friction-induced mechanoluminescence (F-ML) experiment. Figure S4a, b and c depicts spectra of F-ML generated with different forces, from 2 to 30 N, for samples ZnS/CaZnS: Er^3+^ (1%), ZnS/CaZnS: Mn^2+^ (0.1%), Er^3+^ (1%), and ZnS/CaZnS: Mn^2+^ (2%), Er^3+^ (1%), respectively. Note, the summary of the force dependence of the total F-ML intensity is presented in the Manuscript file in Figure 2a-c (bottom). After exposure to light, all tested samples exhibit strong mechanoluminescence (ML). However, similar to the ZnS/CaZnS:Mn material we reported [6], the samples retain their ability to respond to mechanical loading by emitting light even after prolonged storage in darkness.

We also investigated the stability and repeatability of ML under a long sequence of mechanical stimuli. In this experiment, a glass rod was pressed against the sample plate with a force of 10 N and dragged 29 times every 4 seconds at a speed of 6 mm/s over the same area of the sample plate. The ML spectra were recorded every 60 ms. Figure S4 d-f show the integrated ML signal measured for the ZnS/CaZnS:Er³^+^ (1%), ZnS/CaZnS:Mn²^+^ (0.1%),Er³^+^ (1%), and ZnS/CaZnS:Mn²^+^ (2%),Er³^+^ (1%) samples, respectively. It is evident that all three tested samples maintained their ability to exhibit ML without requiring trap recharging through light exposure. The slight decrease in the intensity of successive ML peaks, particularly noticeable at the beginning of a series of rod passes, may be due to compression and displacement of the fine sample powder caused by repeated passes of the rod pressed against the plate. However, after a certain number of rod passes, the ML intensity with successive rod movements stops decreasing and reaches a plateau. This effect can be observed in the images presented in Figure S6, which. shows photographs of three plates with the tested samples, taken after several series of F-ML measurements. The images were taken (through the PMMA plate) under (a) daylight and (b) 365 nm illumination. The areas over which the rod was repeatedly drawn during the F-ML experiments are visible as parallel lines of compressed, thinned powder layer on these plates.


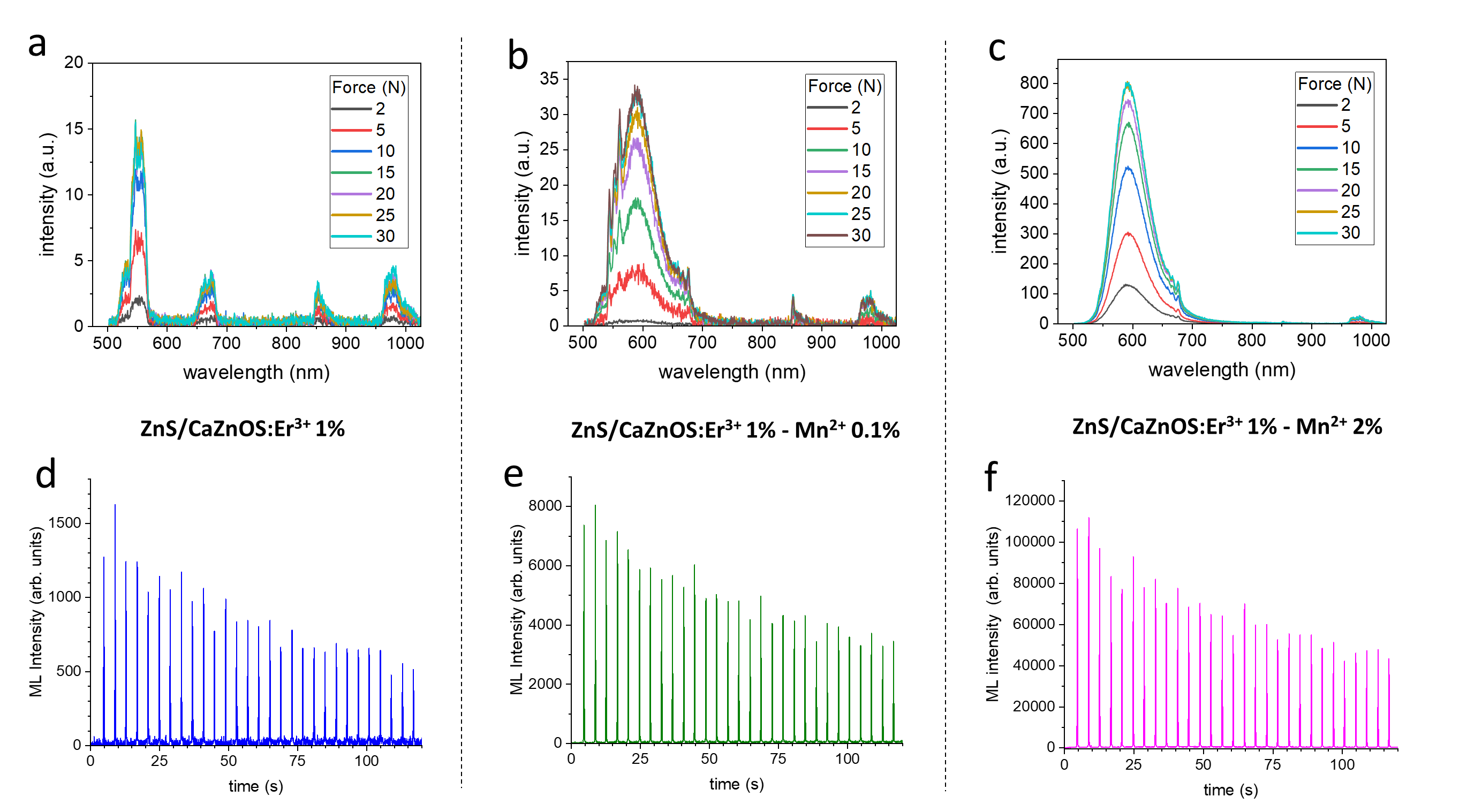


**Figure S4.** a-c) Emission spectra of friction-induced mechanoluminescence (F-ML) generated with different forces for samples ZnS/CaZnS:Er^3+^ 1% (left), ZnS/CaZnS:Mn^2+^ 0.1%, Er^3+^ 1% (center) and ZnS/CaZnS:Mn^2+^ 2%, Er^3+^ 1% (right). d-f) The integrated ML signal measured for the samples ZnS/CaZnS:Er³^+^ 1% (left), ZnS/CaZnS:Mn²^+^ 0.1%, Er³^+^ 1% (center) and ZnS/CaZnS:Mn²^+^ 2%, Er³^+^ 1% (right), where the rod was pressed toward the sample plate with a force of 10 N.

Figure S5a shows the time dependence of the integrated ML signal generated by 150 rod passes on a plate with the ZnS/CaZnS:Mn²^+^ (2%), Er³^+^ (1%) sample. In this test, ML spectra were measured every 0.2 seconds, and the rod was pressed against the plate with a force of 7 N and drawn 150 times across the same part of the sample plate at a speed of 6 mm/s, every 10 seconds. An example excerpt from the long series of the ML spectra measured in this experiment is shown in Figure S5b.


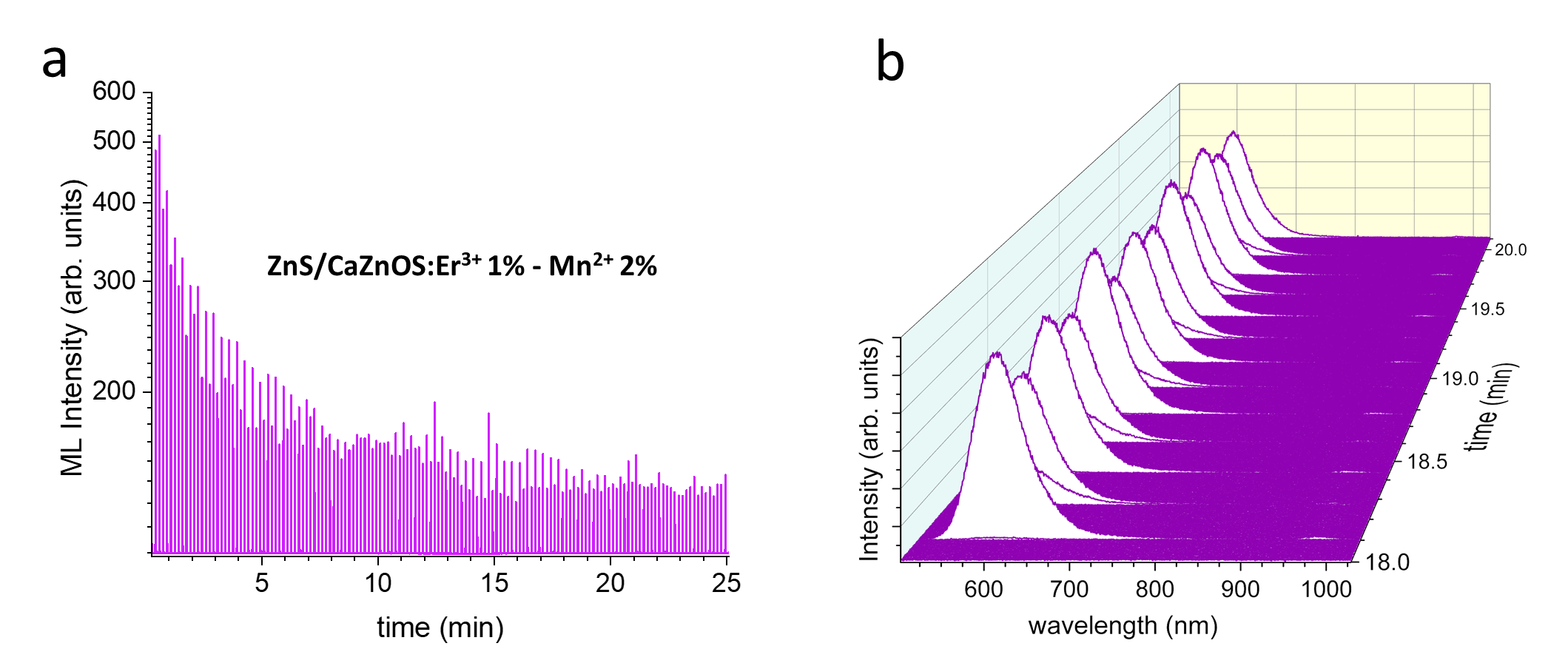


**Figure S5.** Results of the F-ML sustainability test performed on the ZnS/CaZnS:Mn²^+^ (2%), Er³^+^ (1%) sample. a – time dependence of the integrated ML intensity generated by 150 rod movements. The rod was pressed against the sample plate with a force of 7 N. b - An example excerpt from the series of ML spectra.


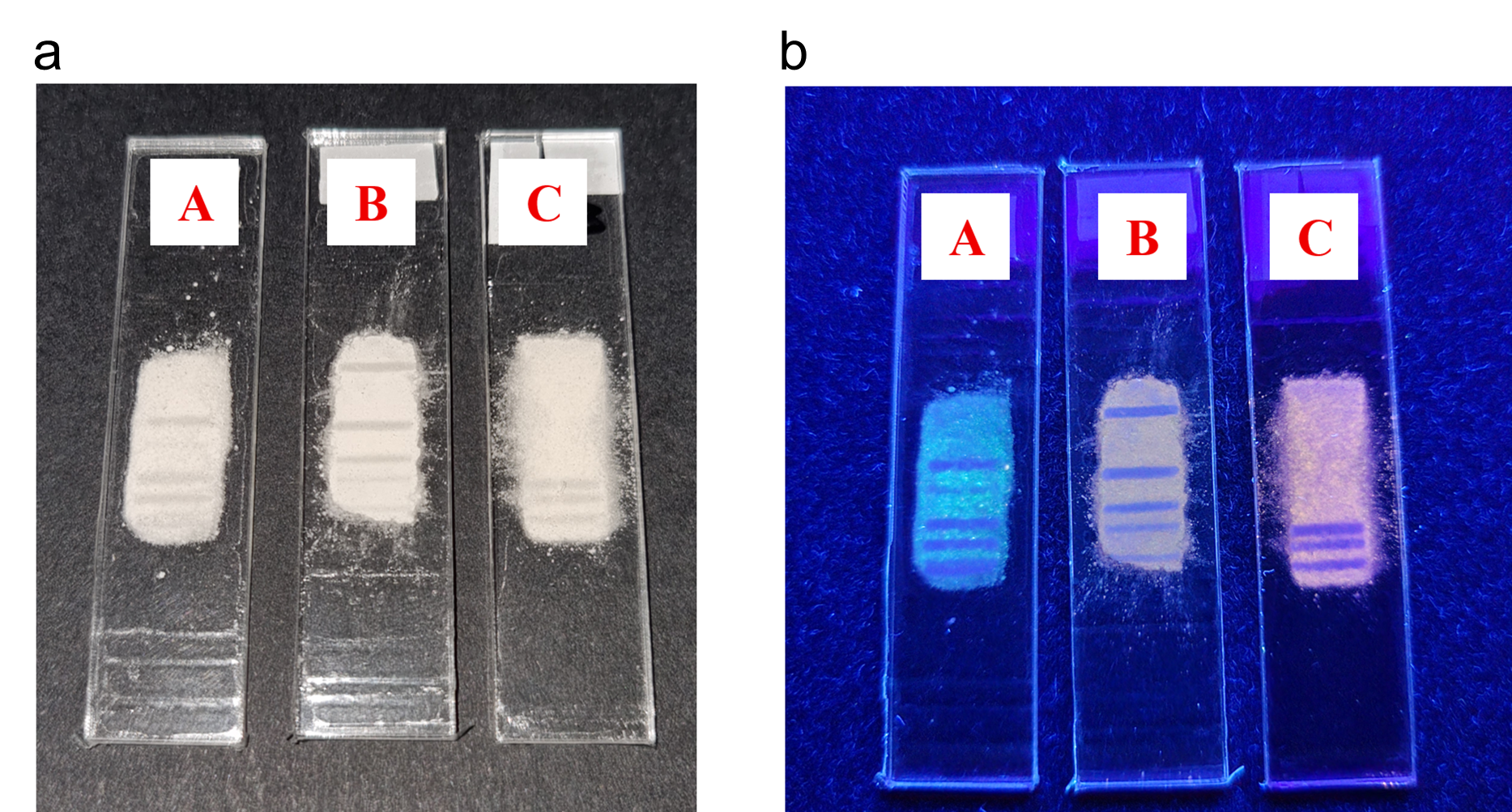


**Figure S6.** Photographs of the plates containing the samples: (A) ZnS/CaZnS:Er³⁺ (1%), (B) ZnS/CaZnS:Mn²⁺ (0.1%), and (C) ZnS/CaZnS:Mn²⁺ (2%), Er³⁺ (1%), after several series of F-ML measurements. The images were taken through the PMMA plates: (a) under daylight (white light) and (b) under 365 nm illumination.

**References**

1 C. D. S. Brites, A. Millán and L. D. Carlos, in *Handbook on the Physics and Chemistry of Rare Earths, volume 49*, 2016, vol. 49, pp. 339–427.

2 S. Balabhadra, M. L. Debasu, C. D. S. Brites, R. A. S. Ferreira and L. D. Carlos, *J. Phys. Chem. C*, 2017, **121**, 13962–13968.

3 M. D. Shinn, W. A. Sibley, M. G. Drexhage and R. N. Brown, *Phys. Rev. B*, 1983, **27**, 6635–6648.

4 T. Zheng, M. Runowski, P. Woźny, B. Barszcz, S. Lis, M. Vega, J. Llanos, K. Soler-Carracedo and I. R. Martín, *J. Alloys Compd.*, 2022, **906**, 164329.

5 J. C. Martins, C. D. S. Brites, A. N. C. Neto, R. A. S. Ferreira and L. D. Carlos, in *Luminescent Thermometry*, Springer International Publishing, Cham, 2023, pp. 105–152.

6 T. Zheng, M. Runowski, I. R. Martín, K. Soler-Carracedo, L. Peng, M.Skwierczyńska, M. Sójka, J. Barzowska, S. Mahlik, H. Hemmerich, F.Rivera-López, P. Kulpi ́nski, V. Lavín, D. Alonso, D. Peng, *Adv. Mater*., 2023, **35**, 2304140,
